# Upconversion rare Earths nanomaterials applied to photodynamic therapy and bioimaging

**DOI:** 10.3389/fchem.2022.1035449

**Published:** 2022-11-17

**Authors:** Thaís K. L. Rezende, Helliomar P. Barbosa, Luiz F. dos Santos, Karmel de O. Lima, Patrícia Alves de Matos, Tayana M. Tsubone, Rogéria R. Gonçalves, Jefferson L. Ferrari

**Affiliations:** ^1^ Laboratório de Desenvolvimento de Materiais Inorgânicos com Terras Raras−DeMITeR, Instituto de Química−(IQ), Universidade Federal de Uberlândia−(UFU), Uberlândia, Brazil; ^2^ Laboratório de Materiais Luminescentes Micro e Nanoestruturados−Mater Lumen, Departamento de Química, FFCLRP, Universidade de São Paulo−(USP), Uberlândia, Brazil; ^3^ Laboratório Interdisciplinar de Fotobiologia e Biomoléculas (LIFeBio), Instituto de Química−(IQ), Universidade Federal de Uberlândia−(UFU), Uberlândia, Brazil

**Keywords:** photodynamic therapy, photoluminescence, rare Earth, nanoparticles, inorganic

## Abstract

Light-based therapies and diagnoses including photodynamic therapy (PDT) have been used in many fields of medicine, including the treatment of non-oncological diseases and many types of cancer. PDT require a light source and a light-sensitive compound, called photosensitizer (PS), to detect and destroy cancer cells. After absorption of the photon, PS molecule gets excited from its singlet ground state to a higher electronically excited state which, among several photophysical processes, can emit light (fluorescence) and/or generate reactive oxygen species (ROS). Moreover, the biological responses are activated only in specific areas of the tissue that have been submitted to exposure to light. The success of the PDT depends on many parameters, such as deep light penetration on tissue, higher PS uptake by undesired cells as well as its photophysical and photochemical characteristics. One of the challenges of PDT is the depth of penetration of light into biological tissues. Because photon absorption and scattering occur simultaneously, these processes depend directly on the light wavelength. Using PS that absorbs photons on “optical transparency windows” of biological tissues promises deeper penetration and less attenuation during the irradiation process. The traditional PS normally is excited by a higher energy photon (UV-Vis light) which has become the Achilles’ heel in photodiagnosis and phototreatment of deep-seated tumors below the skin. Thus, the need to have an effective upconverter sensitizer agent is the property in which it absorbs light in the near-infrared (NIR) region and emits in the visible and NIR spectral regions. The red emission can contribute to the therapy and the green and NIR emission to obtain the image, for example. The absorption of NIR light by the material is very interesting because it allows greater penetration depth for *in vivo* bioimaging and can efficiently suppress autofluorescence and light scattering. Consequently, the penetration of NIR radiation is greater, activating the biophotoluminescent material within the cell. Thus, materials containing Rare Earth (RE) elements have a great advantage for these applications due to their attractive optical and physicochemical properties, such as several possibilities of excitation wavelengths – from UV to NIR, strong photoluminescence emissions, relatively long luminescence decay lifetimes (µs to ms), and high sensitivity and easy preparation. In resume, the relentless search for new systems continues. The contribution and understanding of the mechanisms of the various physicochemical properties presented by this system is critical to finding a suitable system for cancer treatment *via* PDT.

## 1 Introduction

Interaction of light with matter is the basis of photosensitization reactions, which occur after the absorption of light photons by photosensitive compounds and transfer this absorbed energy to neighboring molecules, transforming the energy originated from light into chemical energy (Suppan and Suppan, 2007). In nature, such photosensitization reactions can be beneficial and/or essential for life, for example, the process of photosynthesis in plants uses chlorophyll as a photosensitizer (PS) that captures light energy, converts it into chemical energy, and stores it as carbohydrates and other constituents of plant tissues (Tsubone et al., 2019). However, excessive light exposure can also be associated with harmful effects on living organisms, such as the loss of cell homeostasis promoting cell death, photoaging, mutation, and/or skin cancer (Björn, 2008).

Historical reports describe the use of light as part of treatment in the medical field in Ancient Egypt, Greece, India, and China civilizations until nowadays (Abdel-kader, 2016). Since then, the use of light has become increasingly important as a therapeutic tool and has been obtaining meaningful and positive results in the treatment of various types of cancers (Grzybowski et al., 2016). Nowadays, this process is called Photodynamic Therapy (PDT) and has gained regulatory approval for the treatment of numerous diseases. PDT has been approved in several countries by U.S. Food and Drug Administration (FDA) and/or European Medicines Agency (EMA) to treat age-related macular degeneration (Schmidt-Erfurth and Hasan, 2000; Han et al., 2022), actinic keratosis (Patel et al., 2014; Liew et al., 2020), and several types of cancer: non-melanoma skin cancer (Griffin and Lear, 2016), lung cancer (Maziak et al., 2004), esophageal cancers (Cheng et al., 2018), breast cancer (Banerjee et al., 2020), bladder cancer (Railkar and Agarwal, 2018) prostate cancer (Wang et al., 2019; Fukuhara et al., 2021), brain cancer (Cramer and Chen, 2020), head and neck cancer (Meulemans et al., 2019).

**FIGURE 1:**
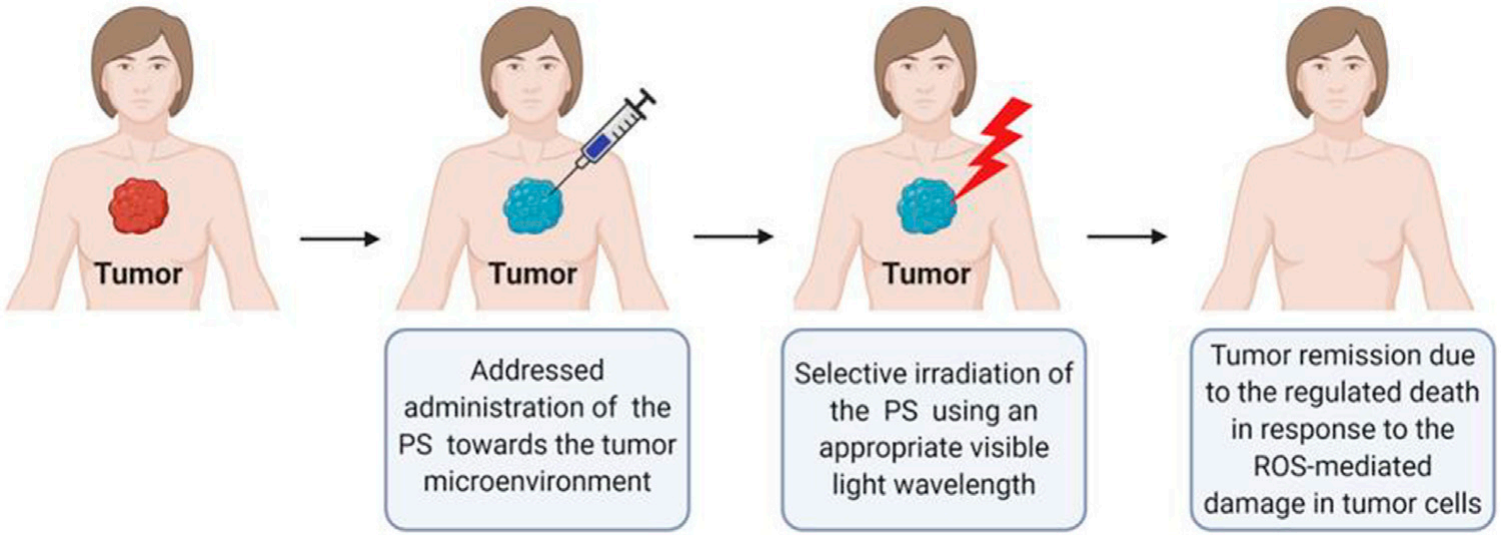
Simplified procedure of photodynamic therapy in patients. Created with BioRenders by authors.

PDT clinical procedure consists of administering an exogenous PS molecule intravenously, intraperitoneally, or topically onto the patient, followed by selectively irradiating the tumor region using an appropriate light source (lasers, light-emitting diodes (LEDs), or lamps), as demonstrated in Figure 1.

The appealing characteristic of PDT is that photodynamic reactions just happen in the region where light reached properly the PS on the tumor. The *in-situ* stimuli of light make PDT destroy target tissue selectively while avoiding unnecessary side effects on healthy tissues. When administered accurately, PDT has none or minimal side effects. It is usually considered less invasive than surgery, more precisely than chemotherapy and, unlike radiation therapy, can be repeated many times (Martins et al., 2021).

Three main components are required for PDT: PS, light on a suitable wavelength, and oxygen inside the tissue. After absorbing the light photons, the electronically excited PS can undergo different photophysical processes through radiative and/or non-radiative transitions. If the light energy absorbed by the PS forms a triplet excited state (^3^PS*) through intersystem crossing (ISC), reactive oxygen species (ROS) can be generated and irreversible damage to the targeted tumor cells. Also, excited state PS can return to the ground state emitting light (radiative transition), which provides another advantage to PDT: the association of therapy and fluorescence imaging at the same time. The fluorescence imaging technique provides high sensitivity, nondestructive, and real-time *in vivo* imaging of organisms (Lin et al., 2021; Yan et al., 2021). The possibility to display a therapeutic protocol co-developed with imaging diagnosis promoted by one specific PS opens a promising avenue in the theranostic field. The good result provided by PDT depends on several factors, such as illumination conditions, PS chemical features, and PS localization. Despite the advantages mentioned above, taking together all favorable characteristics in one PS is not an easy task. Even though FDA and EMA already approved some PSs, such as Photofrin™, Foscan®, and Verteporfin®, none of them presented a “magic bullet” or exhibit all the ideal features of PS (Bacellar et al., 2015). One of the challenges in the search for the ideal PS is the depth of light penetration into tissues. It occurs because most clinically approved PS are excited at the UV or visible light wavelength, which presents limited penetration tissue, thus hampering the application of PDT in the treatment of large or more internal tumors (Fan et al., 2016). Therefore, significant efforts have been developing new PS platforms photoinduced by the near-infrared (NIR) region, whose wavelengths coincide with “optical transparency windows” of biological tissues allowing deeper penetration and less attenuation during the irradiation process.

Luminescent materials that present NIR emissions show a great advantage compared to UV and/or visible emitters. They are based on long-wavelength excitation/emission ranges that can considerably attenuate background emissions, because of the low NIR spectral absorbance for most biomolecules (Frangioni, 2003).

In the last decade, NIR excitation/emission ranges have started to be investigated in quantum dots (QDs) and dyes for sensing, bioimaging, and PDT (Hong et al., 2012; Xu et al., 2016a). A huge range of several QDs has been studied for PDT owing to their interesting characteristics, providing many achievements in cancer treatments. However, the major disadvantage of using QDs is their potential toxic effects, which can be due to the residual organic species on their surface from synthesis, and some toxic metals in their composition such as Cd^2+^, Hg^2+^, and As^2+^. Conventional NIR-II QDs composed of Silver chalcogenides show an interesting potential compared with typical QDs, especially for biological applications (Xu et al., 2016b). Ag_2_S QD-based on natural hydrogel shows NIR-II emissions and low toxicity, as this compound is free of heavy metals and presents antibacterial properties under 808 nm NIR laser irradiation, it kills 99.7% and 99.8% of *E. coli* and MRSA in 4 min, respectively (Du et al., 2022). The embedded Ag_2_S QDs are potential candidates for antibacterial treatments by using PDT therapy. However, this heavy metal-free NIR-II QD class shows relatively low quantum yields in comparison with conventional QDs (Xu et al., 2016b).

In contrast to most QDs and dyes, Rare Earth (RE) doped particles can be interesting candidates for PDT and bioimaging applications due to the wide spectral excitation/emission ranges in the most applied optical biological tissue window (λ ≈ 700–1700 nm), allowing deeper tissue penetration without tissue autofluorescence. Furthermore, RE^3+^ doped materials can have relatively low cytotoxicity, narrow absorption and emission bands, relatively long excited-state lifetimes, and low autofluorescence background in comparison with QDs and dyes (Gai et al., 2014a).

The general aims of this review consist in discussing the pitfalls, advantages, and challenges at molecular basis comprehension of developing PS to PDT from a chemical perspective, with a focus on upconversion RE doped nanomaterials. Through the next sections, a description of PDT fundamentals and the challenges for the rational design of an “all-in-one” PS are discussed.

## 2 Photophysical and photochemical processes in PDT

The basic concept of PDT involves two individually non-toxic components (PS and light) that are combined to induce cellular and tissue damage in an oxygen-dependent manner. In the PDT mechanism, light absorption and energy transfer are the two most important aspects.

The photophysical action behind PDT starts when the PS absorbs photon energy that converts the ground state (S_0_) of the photosensitizer to a more energetic state called the singlet excited state (S_1_). Because this PS excited state S_1_ exhibit a very unstable electronic configuration with unpaired non–parallel electrons, PS may return to the ground state (S_0_) losing energy in the form of heat (internal conversion) or fluorescence (emitting light). From this PS excited state S_1_, it also possibly occurs a process known as intersystem crossing, where the multiplicity of PS is altered to form a more stable triplet excited state (T_1_) with the inversion of one electron spin Triplet excited state (T_1_) has a longer lifetime than the singlet excited state (S_1_) thus this long-lived T_1_ trigger subsequent photochemistry reactions through two main mechanisms: Type I—electron transfer and Type II—energy transfer (Foote, 1991; Baptista et al., 2017; Baptista et al., 2021).

Type I photosensitization reaction involves electron transfer between PS and substrate. This redox reaction can generate a reduced or oxidized substrate giving rise to the corresponding pair of radical ions (PS^•–^ and S^•+^; PS^•+^ and S^•-^). These radical ions participate in a complex set of competitive pathways, which commonly produce reactive oxygen species, such as hydroxyl radical (OH^•^), superoxide ion (O2^-•^), and hydrogen peroxide (H_2_O_2_).

Type II photosensitization reaction refers to energy transfer between the triplet excited state (T_1_) of PS and molecular oxygen (^3^O_2_) to form singlet oxygen (^1^O_2_), a more reactive state of oxygen (Krumova and Cosa, 2016). The amount of energy transfer required for the transition from ^3^O_2_ to ^1^O_2_ is 0.974 eV (or 22.4 kcal/mol or ≈ 1,270 nm). Although most T_1_ of PSs have a set minimum energy of 1.13 eV (or ≈ 1,090 nm), i.e., it is required more energy than 0.974 eV for the irreversible formation of ^1^O_2_ (Algorri et al., 2021).

Both Type I and Type II mechanisms can occur simultaneously, in which the prevalent mechanism will depend on the substrate, the distance between the PS and substrate as well as the oxygen concentration.

**FIGURE 2:**
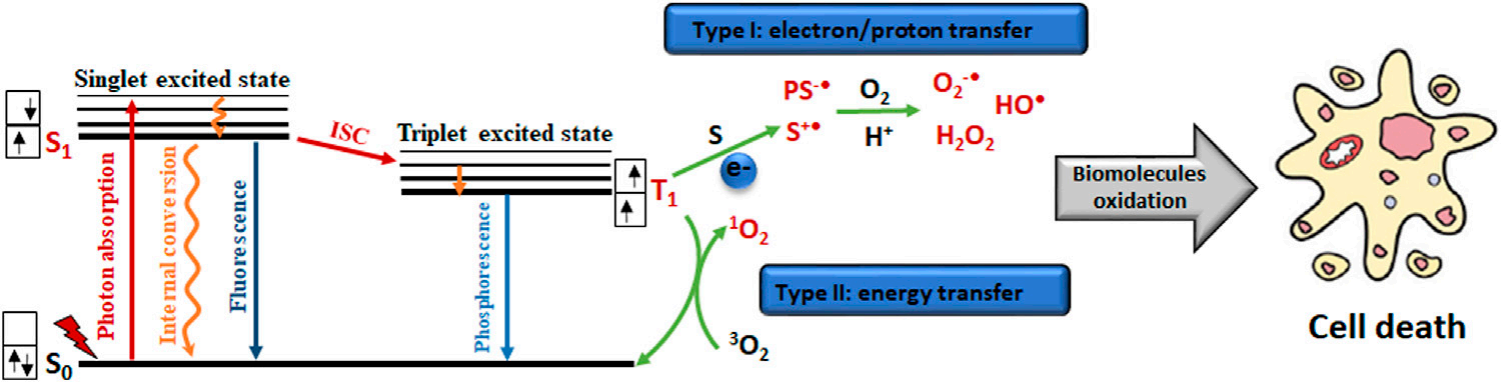
Simplified scheme of the photophysical and photochemical reactions taking place in PDT mechanism, as well as subsequent consequences effects on biochemical molecules and cell death. Adapted from (Gai et al., 2018a).

Generally, the main reaction sites of ROS are electron donor molecules, containing unsaturated carbon-carbon bonds, amines, sulfides, anions, and neutral nucleophiles. That is the reason why both photosensitization mechanisms (Type I and Type II) can damage biomolecules impairing the biological functions of targets such as membranes, proteins, and DNA; thus, compromising cell homeostasis and triggering cell death (Cadet and di Mascio, 2009; Baptista et al., 2021). Figure 2 presents the simplified schemes explained above.

## 3 Light penetration through tissue

**FIGURE 3:**
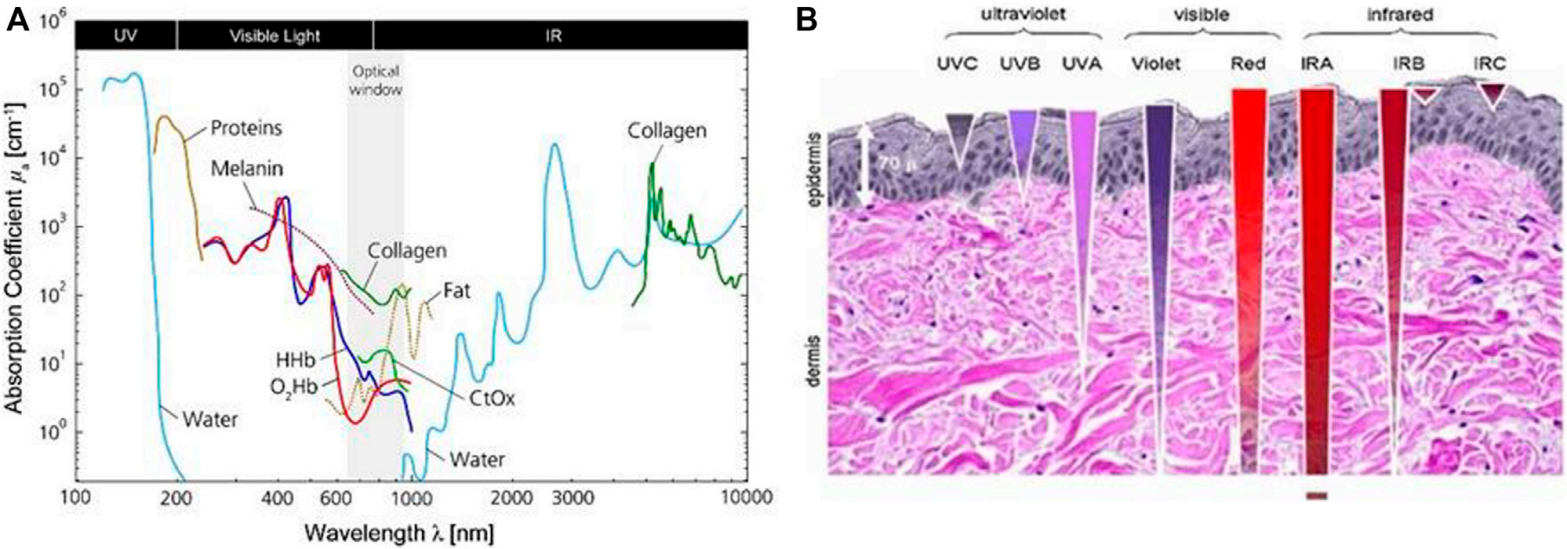
**(A)** Absorption spectra of different endogenous chromophores present in biological tissue. Reprinted from (Scholkmann et al., 2014), Copyright® 2014, with permission from Elsevier. **(B)** Illustrative scheme of light propagation through the tissue. Reprinted from (de Assis et al., 2021c), Copyright® 2021, with permission from Elsevier.

Light penetration depth into the tissue is one critical parameter for the PDT procedure. Since tissues are composed of cells and substances, the interaction between light and tissue depends on their characteristics, such as absorption and scattering properties (Algorri et al., 2021). Endogenous chromophores, including deoxyhemoglobin (HHb), oxyhemoglobin (O_2_Hb), and melanin, exhibit strong absorption of light in the visible spectrum below 650 nm (Figure 3A). This is one of the reasons why blue light penetrates less deeply (< 1.5 mm) through tissue (Protti et al., 2017; de Assis et al., 2021a)—Figure 3B. Typically, the penetration depth of the visible light is only 1–4 mm (de Assis et al., 2021b), which fails to address the requirement of the treatment of solid or deep-seated tumors. Above 950 nm, light is absorbed majority by water as noticed in the spectrum in (Figure 3A). Thus, a larger wavelength (≈ > 1,000 nm) also penetrates less efficiently through tissue (Figure 3B). It means that an optimized “phototherapeutic window” is in the NIR spectral range, between 650 and 950 nm (Yao and Wang, 2014; Protti et al., 2017).

Besides better tissue penetration, this NIR region displays minimum photodamage to cells and low auto-fluorescence in living systems (Ai et al., 2016). However, in conventional PDT, PSs are usually excited in the UV and visible wavelengths. To overcome the drawback of limited penetration depth in traditional PDT systems, several strategies have been applied.

Another recent and intriguing solution for PDT treatment is the use of thermally activated delayed fluorescence (TADF) *via* organic molecule nanoparticles. Zhang et al. (Zhang et al., 2019) presented in their work the use of high-performance metal-free photosensitizers in cancer-mitochondria-targeted based on organic nanoparticles, with the results demonstrating high performance in the ^1^O_2_ production and, consequently, good ROS production.

Fang et al., 2022 (Fang et al., 2022) reported the use of metal-free NIR TADF nanophotosensitizers based on (3,4-bis(4-(diphenylamino)phenyl)acenaphtho(1,2-b)pyrazine-8,9-dicarbonitrile, APDC-DTPA) under two-photon excitation, and the results revealed significant behavior in obtaining the ^1^O_2_ under single and two-photon excitation. Fang et al., 2021 (Fang et al., 2021) published a review describing the different applications of TADF and the many important characteristics associated with their properties. All this desire concerning the search for the best system for application in PDT has made and makes several research groups around the world to delve deeper into the search for new ways to solve problems. One of the great discussions and important results presented by Cui et al., (Cui et al., 2022), is the use of organic radical species that contribute to the search for the improvement of the desired properties, due to the presence of unpaired electrons in its structure, considering the chemical stability of the compounds involved. The physical and chemical properties of the most diverse types of molecules produce signals that can be detected using magnetic resonance, fluorescence, and other techniques. Consequently, this contributes to a wide range of techniques that can aid in the early detection of diseases.

In this sense, upconversion nanoparticles start to draw attention in the fields of phototherapy and bioimaging due to their appropriate nanosize and unique optical properties (Wang et al., 2013; Gai et al., 2014b; Deng et al., 2017; Gai et al., 2018b; Gulzar et al., 2019).

The main advantages of UCNPs that present both NIR excitation/emission are based on the lower autofluorescence of biological tissues, less light scattering, and deeper penetration for *in vivo* imaging and PDT applications. In general, the majority of UCNPs reported in the literature for these fields are based on inorganic matrices with high chemical stability, good optical, thermal, and mechanical properties, cellular biocompatibility, and reasonable stability in biological mediums. These features make this luminescent material class a versatile and promising alternative for PDT.

The synergistic combination of UCNPs and PSs can enhance the scope and applicability of PDT in treating some non-superficial tumors. In the next sections, designing ideas and principles of UCNP-PS will be discussed in detail for some typical examples to be used in phototherapy.

## 4 Photosensitizers

PS is a key factor element in PDT and the ideal PS requires a series of characteristics such: 1) accumulate more in the diseased/target tissue rather than the healthy cells; 2) extremely low dark toxicity (i.e., activated only upon irradiation); 3) strong absorption (≥ 20000–30000 M^−1^cm^−1^) at the phototherapeutic window (∼650 – 950 nm); 4) large singlet-to triplet intersystem crossing efficiency (high quantum yield of ROS); 5) be a single chemical compound with a known and constant composition; 6) exhibit good solubility in water (low aggregation) or form stable drug formulation; 7) chemical and physical stability when in biological medium containing serum or plasma or other biomolecules; and 8) rapid clearance to avoid prolonged phototoxic side effects. Combining all these characteristics in a single PS is extremely challenging, and it is not surprising that several researchers still invest efforts to develop compounds that correspond to as many of the required criteria as possible. Since a large number of PSs have been explored for PDT, the next subsections will discuss the “traditional PS” designed for conventional PDT and subsequently, the description of how upconversion nanomaterials-based PSs may help to overcome problems related to some limitations found in the PDT technique.

### 4.1 Traditional PSs

The first class of PS for PDT cancer treatment exhibits tetrapyrrolic chemical structure as the main portion of chromophores. These tetrapyrrolic structures are present in several important biomolecules, such as heme, chlorophyll, and bacteriochlorophyll. That is the main reason why tetrapyrrole structures were named “pigments of life” (Battersby, 2000).

**FIGURE 4:**
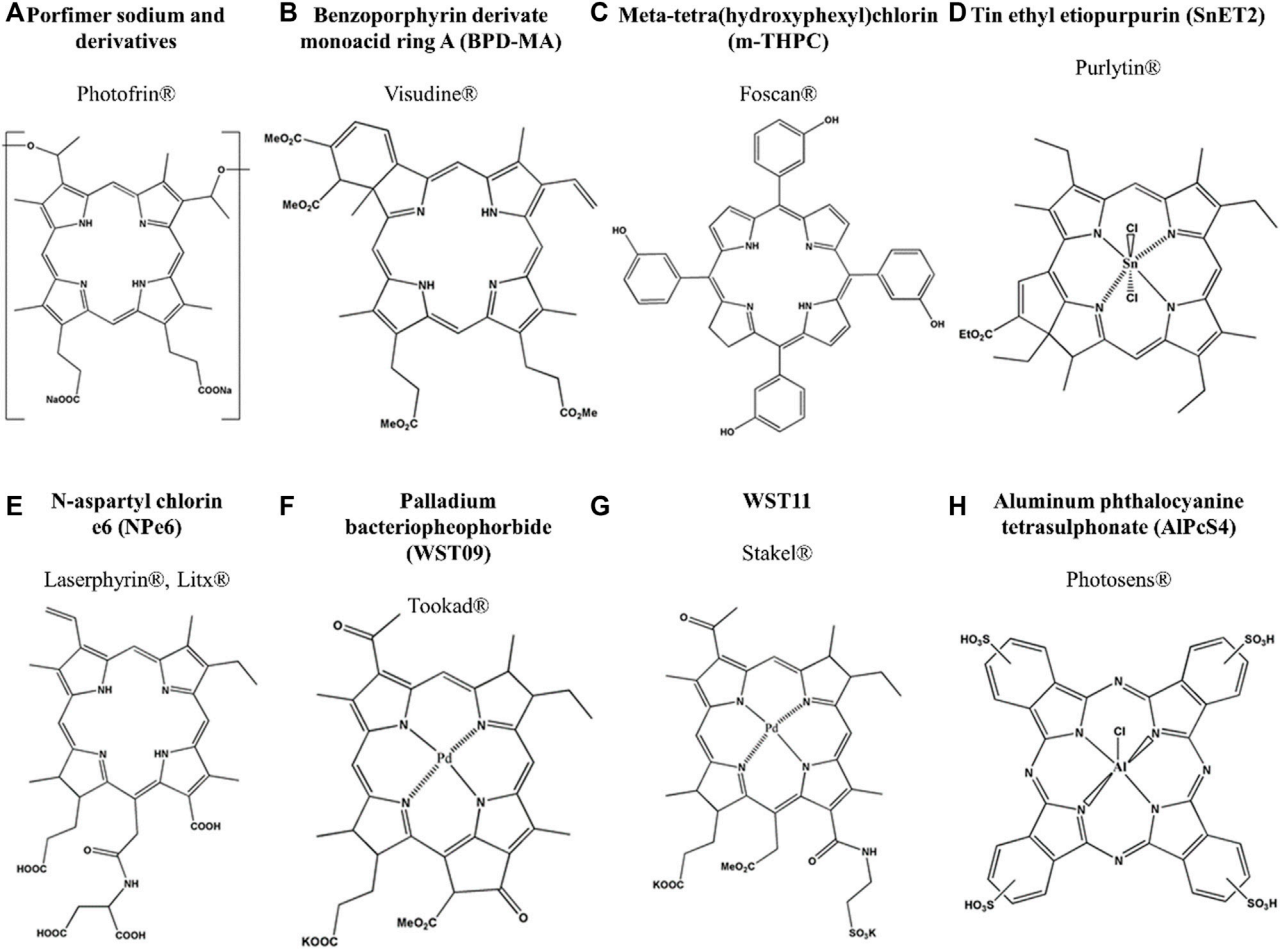
Structure of approved PS for PDT by regulatory agencies. **(A)** Photofrin, **(B)** Visudine, **(C)** Foscan, **(D)** Purlytin, **(E)** Laserphyrin, **(F)** Tookad, **(G)** Stakel and **(H)** Photosen.

Around 1970 Dr. Thomas Dougherty and colleagues tested PDT technique using a porphyrin mixture soluble in water known as “hematoporphyrin derivative” (HpD) in several patients with primary or secondary skin tumors (Gold, 2011). The successful demonstration of PDT efficacy encouraged the commercial development of Photofrin, a mixture of porphyrin dimers and oligomers isolated from HpD (Abrahamse and Hamblin, 2016). Even though Photofrin is the most PS used for PDT, its manufacture has some limitations in clinical application due to low chemical purity (consisting of a mixture of 60 molecules), or poorly light tissue penetration because of low-intensity photon absorption in the region of 630 nm. Also, PDT treatment using Photofrin reported high accumulation in the skin promoting hypersensitivity to light for several weeks (Chatterjee et al., 2008; Zhang et al., 2017). These disadvantages presented by first-generation photosensitizers (porphyrin-based PS) created the necessity to investigate new compounds and initiated the development of second-generation photosensitizers (Chatterjee et al., 2008; Zhang et al., 2017). Thus, second-generation PS includes compounds such as porphyrins (with defined chemical structures and rapid clearance), chlorins, bacteriochlorins, pheophorbides, and phthalocyanines (Yoon et al., 2013; Moriwaki et al., 2018). Second-generation PS is characterized by chemical purity, higher singlet oxygen generation, and better penetration deeply into tissues due to their maximum absorption in the wavelength range of 650–800 nm. Besides that, fewer side effects, greater selectivity for tissue cancer cells, and faster elimination from the body (Kwiatkowski et al., 2018). However, the main disadvantage of second-generation PS is the low water solubility, which is a significantly limiting factor in its administration and again promotes the necessity of the search for new PS or nanoparticles to carry in an aqueous environment (Castano et al., 2004). Third-generation photosensitizers contain first- and second-generation PS conjugated to, or packaged within carrier molecules such as antibodies, liposomes, and polymers in order to improve selectivity and bioavailability (Josefsen and Boyle, 2008). To reduce damage to healthy peripheral tissues, some modifications are being used such as: (I) combinations of second-generation PS with molecules focused on the target receptor; (II) combinations of PSs with low-density lipoprotein (LDL), since proliferating tumor cells need more cholesterol for cell membrane formation; (III) conjugation of PS with a monoclonal antibody directed to the specific antigen of certain cancer cell; and (IV) the use of surface tumor markers, such as growth factor receptors, transferrin receptors, or hormones (e.g., insulin) (Kwiatkowski et al., 2018). Despite significant progress through PS design, up to nowadays, there are no PS with “all-in-one” ideal features. Figure 4 presents the structure while Table 1 shows the main properties of PS already approved by regulatory agencies.

**TABLE 1:** Properties of approved PS for PDT by regulatory agencies (Ormond and Freeman, 2013), (Hamblin, 2020).

Compound	Trademark	λ(nm), ε (M^−1^cm^−1^)	ϕ_Δ_	Excipient formulation	Approval information	Main disadvantages	Reference
Porfimer sodium and derivatives (Figure 4A)	Photofrin®	632 (3,000)	0.89	Dextrose or NaCl injection	Bladder cancer (Canada, 1993), Esophageal cancer (FDA, 1995), Lung cancer (FDA, 1995)	Significant skin photosensitivity and low absorption at the phototherapeutic window	(Ormond and Freeman, 2013) and (Hamblin, 2020)
Benzoporphyrin derivate monoacid ring A (BPD-MA) (Figure 4B)	Visudine®	689 (34,000)	0.84	Liposomal formulation containing 5% dextrose	Age-related macular degeneration (FDA, 2000)	Eyes will be extra sensitive to light (sunlight and bright indoor lights), 5 days after receiving an injection of verteporfin	(Ormond and Freeman, 2013) and (Hamblin, 2020)
Meta-tetra(hydroxyphexyl)chlorin (*m*-THPC) (Figure 4C)	Foscan®	652 (35,000)	0.87	40% ethanol and 60% propylene glycol	Neck and head cancer (EMA, 2001)	Photosensitive for up to 20 days after initial illumination	(Ormond and Freeman, 2013) and (Hamblin, 2020)
Tin ethyl etiopurpurin (SnET2) (Figure 4D)	Purlytin®	664 (30,000)	--	Cremophor-EL emulsion	Breast adenocarcinoma, basal cell carcinoma, Kaposi´s sarcoma, age-related macular degeneration	Produce a photoreaction 7–14 days post-administration	(Ormond and Freeman, 2013) and (Hamblin, 2020)
N-aspartyl chlorin e6 (NPe6) (Figure 4E)	Laserphyrin®, Litx®	664 (40,000)	0.77	---	Lung cancer (Japan, 2004)	---	(Ormond and Freeman, 2013) and (Hamblin, 2020)
Palladium Bacteriopheophorbide (WST09) (Figure 4F)	Tookad®	763 (88,000)	0.50	Cremophor EL	Prostate cancer	---	(Ormond and Freeman, 2013) and (Hamblin, 2020)
WST11 (Figure 4G)	Stakel®	---	---	---	Prostate cancer (EMA, 2017)	---	(Ormond and Freeman, 2013) and (Hamblin, 2020)
Aluminum phthalocyanine tetrasulphonate (AlPcS_4_) (Figure 4H)	Photosens®	676 (200000)	0.38	---	Stomach, skin, lips, oral cavity, tongue, breast cancer (Russia, 2001)	---	(Ormond and Freeman, 2013) and (Hamblin, 2020)

Note that approved PS, commercially available still needs improvements, light penetration still acts as a limiting factor of PDT, in deep tissue in clinical practice. FRET-based indirect excitation of PSs, such as upconversion nanoparticles, may open new avenues for deep PDT.

## 5 Upconversion nanomaterials (UCNPs)-based PSs for PDT

RE elements, specifically trivalent lanthanide ions (Ln^3+^), present several energy levels of 4f configurations which show singular and fascinating optical properties. Owning intermediate energy levels, Ln^3+^ can give out several emissions *via* various energy transfer possibilities. The majority of photoluminescent emitters usually follow the principle of the Stokes law, which is simply states excited by photons with higher energy than the emitted ones or, in other words, that output photon energy is weaker than input photon energy (Auzel, 2004).

**FIGURE 5:**
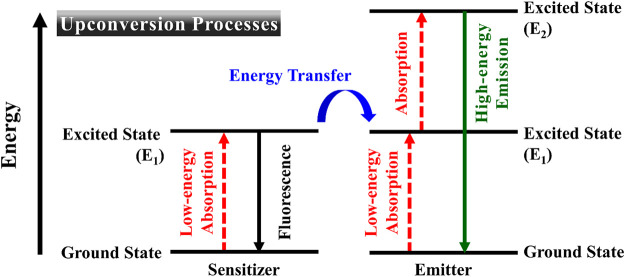
Upconversion processes involving low-energy photons absorption and energy transfer.

In contrast to the conventional photoluminescence process of generating a Stokes shift (Beija et al., 2009), upconversion luminescence is generated from the absorption of two or more continuous-wave (CW) low-energy photons (longer wavelength) and releasing one high-energy emission photon (shorter wavelength) by sequential aborption and energy-transfer processes from the called sensitizer to the emitter ion (Auzel, 2004). Figure 5 presents these cited upconversion processes.

This anti-Stokes photoluminescence process results in low background noise, large tissue penetration depth, and low photo-damage in bioimaging applications and PDT, for example. Due to these characteristics, RE ions are being studied for application in PDT.

Notwithstanding the efficiency therapeutic of photodynamic therapy is limited due to the low ^1^O_2_ yield quantum and restricted energy converted from UCNPs to the PS molecules, several studies have been published in the literature showing significant efficiency of UCNPs coupled with other materials *in vitro* and *in vivo* assays. An important work to be mentioned is the production of UCNPs coupled with graphene carbon dots (Zhang et al., 2018), where the high production of ^1^O_2_ is discussed due to the efficient energy transfer from UCNPs to graphene carbon dots with a specific target in the cells’ mitochondria, concluding a high efficient PDT in breast cell line model. On the other hand, Cai, L. (2022) (Cai et al., 2022) developed a study to treat tongue cancer using nanoparticles based on GdOF tridoped with Er^3+^, Yb^3+^ and Ce^3+^ functionalized with dihydroartemisinin (DHA) and target antibody (RENP-DHA-Cap). In this work, the authors concluded that *in vivo* and *in vitro* experiments, the functionalized nanoparticles provide excellent results for *in situ* imaging and treatment of tongue cancer, prospecting an efficient theranostic platform.

Regarding the pros and cons of the use of materials containing rare earths, some of these characteristics are presented in Table 2 below.

**TABLE 2:** Advantages and disadvantages of RE – doped nanoparticles for application in PDT.

Material	Pros	Cons
Rare Earth – doped nanoparticles	- High chemical and photochemical stability - Offer a wide range of emission profiles depending on the appropriated dopant(s) (Skripka et al., 2019) - Sharp emission spectra - Long lifetime photoluminescence - Reduction of light scattering - Tunable emission - Low toxicity - Good biocompatibility - Non–invasive detection (Qu et al., 2020), (Qu et al., 2020)	- Low quantum yield of the upconverting process - High quenching of emission in aqueous medium - Whereas other materials such as organic dyes and Quantum Dots are easily reproducible, systematic routes design is needed for synthetic and consequently surface modification of rare earth materials (Wani and Majeed, 2017)

## 6 Strategies to improve upconversion in rare Earth materials

Many strategies have been developed to enhance the intensity of upconversion, including doping and co-doping, designing different host matrices, modulating particle size and morphology, varying the synthesis, and others.

The co-doping is a strategy to enhance the UC processes since an ion can act as a sensitizer, strongly absorbing energy in the IR region and transferring effectively to an activator ion, which will emit in the UV/Visible region (as shown in Figure 5). Mokoena and collaborators (Mokoena et al., 2021) co-doped the Ba_5_(PO_4_)_3_OH material with Er^3+^ and Yb^3+^ ions. Under 980 nm excitation, it exhibits red and green emissions, both enhanced by the co-doping. Yin and others (Yin et al., 2017) report considerably enhance UC intensity by the Yb^3+^ codoping Y_2_Ti_2_O_7_:Er^3+^ under 1,550 nm and 980 nm excitation. Van and others (Van et al., 2021) reported how the doping with Mo^6+^ increased 70 times the green intensity emission of HA:Er-Yb phosphor. Rong and collaborators (Rong et al., 2020) studied the detection of diazinon in food to the fluorescence quenching of β-NaYF_4_: Yb, Er UCNPs, and graphene oxide.

Although the cooping enhances the UC phenomenon, the concentration of the doping ions must be designed in a way that the distance between the sensitizer and activator ions is not so great as to impede the effective energy transfer, and not so close that non-radiative decays such as cross-relaxation processes and energy migration occur (Yang et al., 2002; Wen et al., 2018) With this in mind, many researchers have been studying the concentration ratio between dopant ions to improve the upconversion process. Guo and others (Guo et al., 2017) studied the concentration quenching of Yb^3+^/Tm^3+^ in UC systems and unusually intense emissions for the NaYF_4_:Yb/Tm material. Their UV and blue emission intensity enhanced around 56 times and 8 times, respectively, when the concentration of Yb^3+^ increased from 20 to 80 mol%. The NaF/RE (RE: Yb, Tm) concentration ratio and its effects on NaYbF_4_:Tm^3+^ microrods were also studied. It was found that the size and morphology could be controlled by the molar ratio used, besides the intense UV/Visible emissions obtained under 980 nm excitation.

One possible way to avoid the quenching of concentration is coating the particles with a surface such as silica (SiO_2_). In the work of Kowalik and others, the authors use the approach of coating the RE ion doped NaYF_4_ nanoparticles with silicon oxide shell (NaYF_4_:20%Yb,0.2%Tm@SiO_2_) for applications in the medical field (Kowalik et al., 2021). The core@shell NPs were prepared *via* homogenous solution co-precipitation followed by the sol-gel method. The UV-Vis emission bands (475, 646, 697, and 803 nm) arise from the 4f-4f levels of Tm^3+^, after receiving the energy transferred from the Yb^3+^ ions. The upconversion observed in the 803 nm band can be used in applications such as bioimaging, as well as the 475 nm to ROS generation in PDT.

Vlasenko and Bakhmetyev (Vlasenko and v Bakhmetyev, 2021) used the microwave heat treatment of Y_2_O_3_:Eu (with and without Aerosil) to improve the crystal, enhancing the luminescence bands’ intensities. They compared these samples with the Rapid Thermal annealing (RTA) method. The MW annealed samples presented crystallites around 26 nm meanwhile the RTA samples presented 12 nm. By adding pyrogenic silica (Aerosil), the MW annealing allowed the yttrium and silicon oxides to react with each other. On the contrary, for the RTA samples, the Y_2_O_3_ and SiO_2_ do not react with each other at the same 800°C. Although the addition of Aerosil in the RTA samples leads to a decrease in the luminescence intensity, it has enhanced the dispersion, which is required for the use in PDT. For the MW annealed samples, the intensity of the peaks at 590, 600, 621 (the most intense band), and 700 nm increased.

There are several tools to improve the efficiency of rare earth materials in PDT, such as porous structure coatings, ligand exchange, and surface polymerization. As discussed previously, the use of mesoporous silica shell can enhance the upconversion properties of rare earth materials and also provides biocompatibility, easy surface modification, and increase the photosensitizer loading capability (Qian et al., 2009).

The mesoporous silica preserves encapsulated from the biological environment, and this kind of coating is considered one of the most important drug delivery systems for hydrophobic drugs. There are several strategies to produce gated porous silica with controlled drug release and improved NP accumulation around the tumor tissues (Gisbert-Garzarán and Vallet-Regí, 2020).

Amine functionalization with 3-(aminopropyl) triethoxysilane (APTES) is one process that supports binding metal ions through the terminal NH_2_ groups. This functionalization alternative is used for UCNPs association with noble metallic NPs, such as gold or silver, to improve the UCNP luminescence efficiency by surface plasmon resonance (SPR) (Ge et al., 2013). In particular, gold nanoparticles present high biocompatibility, and thermal and non-thermal paths for reactive oxygen species formation (Huang and El-Sayed, 2010).

Güleryüz and co-authors (Güleryüz et al., 2021) have reported the use of dual photosensitizers (ZnPc/MC540) with mesoporous silica and gold NPs coated upconversion nanoplatforms to enhance PDT treatment for prostate cancer. This platform exhibits high biocompatibility and considerable production of ROS after 5 min of NIR light excitation.

UCnPs@AgBiS_2_ core-shell nanoparticles present combined photothermal/photodynamic therapy against malignant tumors. Particularly, hexagonal NaYF_4_: Yb/Er/Nd@NaYF_4_: Nd cores show intense upconversion when the doping concentration of Nd ions was fixed at 1% in the inner core, exciting the AgBiS_2_ shell to produce ROS for photodynamic therapy (PDT) of cancer cells. The AgBiS_2_ coating exhibits great chemical stability and significant cancer-cell-specific cytotoxicity. The potential cross-relaxation process between the continuous energy band of AgBiS_2_ and the Nd^3+^ energy levels promote the excellent photothermal conversion and ROS production of this core-shell system (Chu et al., 2022).

The association of UCNPs with quantum dots (QDs) presents promising multifunctional nanoplatforms acting on tumor-target chemotherapy-photodynamic therapy. Cao and collaborators have published a MoS_2_ QDs decorated NaYF_4_:Tm^3+^, Yb^3+^ system which converts NIR to visible light, which can stimulate MoS_2_ to generate abundant ROS through FRET. The nanoplatform was functionalized with polyethylene glycol (PEG), and folic acid (FA) endowed UCNP/MoS_2_ with good physiological stability, biocompatibility, and FA-mediated tumor targeting (Cao et al., 2022).

## 7 NaYF_4_:RE^3+^ upconversion nanomaterials applied in PDT

Rare Earth doping requires host materials with low phonon energies in order to enable efficient upconverter emission. Low multiphonon relaxation rates and non-radiative losses are ensured by the low-energy phonons. With phonon energies equal to 144, 172, and 260 cm^−1^, respectively, chlorides, bromides, and iodides are theoretically suitable host matrices for upconverter emission. These nanoparticles, on the other hand, frequently present low chemical stability and are prone to deliquescence in ambient settings. Despite their chemical stability, oxides have high phonon energies of approximately 600 cm^−1^. They are not good to host matrices for UC phenomena because they make the activator's multiphonon relaxation rates comparable to the radiative emission rates. However, phonon help may be required at times throughout the UC process, therefore there is a compromise between phonon characteristics and UC efficiency. The most efficient and stable host matrices for RE^3+^ doping are fluorides. Due to their low phonon energies (350 cm^−1^) and desirable chemical stability, these RE^3+^-doped nanomaterials can achieve powerful upconversion emission for a variety of applications, such as photodynamic treatment. NaYF_4_ has long been known as the most effective nanostructure for the UC phenomenon. In addition to phonon energy, the crystalline structure of the host matrix is important for improving upconverter efficiency. In comparison to cubic-phase NaYF_4_ (alpha) nanomaterials, hexagonal-phase NaYF_4_ (beta) nanomaterials have an order of magnitude higher upconverter efficiency. The variable crystal fields around RE^3+^ ions in the host lattice can be directly attributed to the phase-dependent optical characteristic (Auzel, 2004; Wang et al., 2015; Zhang, 2015).

On the topic of PDT, investigation on UCNP has made significant development in recent years. For instance, the surface of the NaYF_4_:Yb^3+^/Er^3+^ UC luminescence material was covered with a layer of silica, and then the photosensitizer MC540 was compounded on top of the silica, followed by the usage of an antibody that targets cancer cells. The PDT test of mice *in vivo* was the first-time upconverting luminescence material was used. The photosensitizer Ce6 was placed onto the polyethylene glycol-modified upconversion luminescence material, which was subsequently injected into mice that had been inoculated with breast cancer. The findings of the experiment revealed that 70% of tumors were eradicated and did not reappear (Wu et al., 2022).

Photosensitizers and other light-excitable chemicals have the property of being harmless when not stimulated but exhibiting cytotoxicity when triggered, allowing them to effectively act on cancer cells in living creatures while minimizing tissue and organ damage (Xu et al., 2017). NaYF_4_:Yb^3+^/Er^3+^ UCNPs with a hexagonal lattice can be employed for upconversion photodynamic treatment in animals. The particles’ exterior layer is covered with a chlorin e6 photosensitizer-containing polyethylene. Under NIR excitation, a glycol polymer can generate singlet oxygen, which can kill tumor cells. However, because the particles lack targeting ligands, they are unable to select specific tumor cells, and their upconversion efficiency is modest, limiting the benefit of dynamic therapy. Coating the surface of NaYF_4_:Yb^3+^/Er^3+^ with mesoporous silica containing tumor-targeting ligands can compensate for the shortcomings of UC photodynamic treatment in the body, improve target cell selectivity, and increase UC efficiency (Zhang et al., 2007).

To facilitate the PDT for deep-tissue cancers, improve selectivity and biocompatibility, UCNPs suitable for biological application can be synthesized using a variety of chemical methods, with further surface functional modification by photosensitizers, hydrophilic groups, targeting agents, specific antibodies, and so on. PEI (polyethyleneimine) contains a huge number of amino groups with strong reactivity and polarity, as well as a huge number of positive charges. As a result, surface modification of the NaYF_4_:Yb^3+^/Er^3+^ nanoparticles with PEI could increase not only their hydrophilicity but also their reactive activity, allowing for further functionalization. The positive charge on the PEI surface can also cling to negatively charged residues on cellular surfaces and penetrate *via* endocytosis to promote medication uptake, a process known as the enhanced permeability and retention effect (EPR) (Ding et al., 2022). At relatively low temperatures, NaYF_4_:20%Yb^3+^,2%Er^3+^@PEI UCNPs were synthesized using a one-step hydrothermal preparation method. Photosensitizer Ce6 and anti-EpCAM, a highly expressed monoclonal antibody in hepatocellular carcinoma cancer stem cells, were linked to UCNP surfaces *via* an amide linkage formed by the carboxyl from Ce6 or anti-EpCAM and abundant amino group arising from PEI, resulting in Ps-Ce6 and anti-EpCAM-UCNPs-Ce6 nanoparticles. Under NIR excitation (980 nm), *in vitro* experiments revealed that the system has good biosafety, targeting, and PDT tumor therapy effects (Ding et al., 2022).

To realize the NIR light switch cascade reaction triggered by particular microRNA and accurate PDT for early cancer, Zhang and collaborators (Zhang et al., 2020) developed an amplifier with multiple UC luminescence made up of photo-caged DNA nano-combs and NaYF_4_:Tm,Yb,Gd@NaYF_4_:Nd, Yb (x_Tm_: 0.5; x_Yb_: 30; x_Gd_: 10; x_Nd_: 30; x_Yb_: 10%) UCNPs sensitized with IRDye® 800CW. Under 808 nm source excitation, the produced ultraviolet light shuts off the “photozipper” causing the microRNA response to cascade hybridization reaction. To carry out efficient PDT, this triggers the photosensitizer coupled to various hairpins to form ROS under the blue light emitted simultaneously. The amplifier demonstrated good serum stability, high reactive oxygen species generation controllability, strong cancer specificity, and sensitivity to particular microRNA expression. *In vivo* and *in vitro* investigations revealed significant inhibition of cell proliferation, as well as the potential to cause tumor cell death and tumor growth reduction.

Li and collaborators (Li et al., 2020) developed a form of intracellular cathepsin B (CAB) upconversion nanoprobe that can predict therapeutic impact in real-time. The multishell UCNPs NaYF_4_:Gd@NaYF_4_:Er,Yb@NaYF_4_:Nd, Yb were covalently modified with an antenna molecule 800CW for luminescence narrow bands enhancement under NIR excitation. The emission spectra exhibited intense narrow bands located at 520, 540, and 650 nm arising from ^2^H_11/2_→^4^I_15/2_, ^4^S_3/2_→^4^I_15/2_, and ^4^F_9/2_→^4^I_15/2_ transitions of Er^3+^, respectively. Besides, the system presents photosensitizer Rose Bengal (RB) for PDT and Cy3 for therapeutic effect prediction. The fluorescence intensity ratio of Cy3 over UCNPs (FI583/FI540) is assessed for self-corrected therapeutic effect prediction since UCNPs emission at 540 nm remains stable during the peptide cleavage process, which serves as an internal reference for Cy3 emission correction. The self-corrected upconversion nanoprobe has a lot of promise in terms of precision tumor treatment.

Sun and coworkers (Sun et al., 2019) designed multi-functional lanthanide-doped upconversion nanocomposites that can not only offer temperature feedback in the photothermal therapy (PTT) process but also perform PDT for a synergistic impact in tumor therapy. Mesoporous SiO_2_ was changed on the surface using NaYF_4_:Yb, Er UCNPs, and photosensitizer Ce6 molecules, which were excited by Er^3+^ red emission at 980 nm power source. The temperature of the PTT site may be monitored by measuring the ratio of I_525_/I_545_ of green emissions, especially within the physiological range, since Cit-CuS NPs were further connected to the surface of the composite as a photothermal conversion agent. They evaluated the dual-modal therapeutic impact *in vitro* and *in vivo*, respectively, based on the guidance provided from spectrum investigations, and found promising findings. Furthermore, these rare earths doped nanoparticles exhibit a high scintillation luminescence that may be utilized for X-ray-induced photodynamic therapy, which is a hot topic since this novel therapy can be used to treat both deep and superficial cancers.

Tsai and coworkers (Tsai et al., 2021) investigated, synthesized, and characterized photo- and redox-responsive polymersomes for cancer treatment, using the double emulsion process to build polymersomes from the synthesized amphiphilic diblock copolymer poly(ε-caprolactone)- o-nitrobenzyl (ONB)-SS-poly(methacrylic acid) (PCL-ONB-SS-PMAA). A responsive ONB ester was used to connect the two polymeric chains, which was followed by a GSH-responsive disulfide linkage (SS). UCNPs with hydrophobic cores and shells [NaYF_4_:Yb, Tm (core)/NaYF_4_ (shell)] and doxorubicin (DOX) were enclosed into the polymersomes at the same time during construction. The hydrophilic core was encased with DOX, and the hydrophobic core-shell UCNPs were loaded into the hydrophobic bilayer. Under NIR excitation at 980 nm, UCNPs emit *in situ* UV light about 365 nm, causing the ONB bond photorupture. It increased DOX release for chemotherapy by combining ONB linkage-induced photorupture and GSH disulfide cleavage. Under the guidance of a 980 nm power laser, polymersomes comprising core-shell UCNPs and DOX (UCNP-PNSP@DOX NPs) were cytotoxic against three lung cancer cell lines (A549, CR-5802, and HEL-299 cells). Under 980 nm laser excitation, UCNP-PNSP@DOX NPs reduced tumor development in A549 tumor-bearing mice compared to those without laser irradiation and those treated with free DOX.

However, the main obstacle with 980 nm excitation is that it has insufficient energy to activate PS, necessitating the use of a strong light source. Furthermore, most UNCPs that use Yb upconversion at 980 nm have severe drawbacks since this NIR excitation wavelength coincides with water absorption in the first biological window. As a result, UCNPs that can be stimulated at wavelengths other than 980 nm, such as 808 nm or 1,064 nm, will be more useful in biological applications. Martinez and collaborators (Martínez et al., 2020) prepared and characterized photosensitizer-polymer-modified UCNPs that can be excited by 808 nm for application in PDT in this quest. Lanthanide-doped core@multishell UCNPs with a size of roughly 60 nm were prepared in organic solvents to obtain NaYF_4_:Yb18%,Er2% at NaYF_4_:Yb10% at NaNdF_4_:Yb10% at NaYF_4_:Yb10% luminescent material with a highly efficient 808 nm to visible wavelengths conversion. Both 980 nm and 808 nm wavelengths may be used to excite these UCNPs, and their narrow emission bands remain the same at 522, 542, and 657 nm. RB and Ce6 PSs were added to these UCNPs. Furthermore, they have been shown to be localized in mitochondria and lysosomes, and these mitotracker/lysotracker dyes were applied to stain these organelles.

Drug delivery, diagnostics, and theranostics are just a few of the biological and healthcare applications for UCNPs. These applications rely on biocompatible materials that provide no or no risk to human cells, tissues, and organs. The issues of reproducible synthesis, effective control over NP size and shape, creation of a biocompatible surface design with biorecognition features for labeling target locations, light activation, theranostics, and drug administration are all involved with the translation of UCNPs. Some of the most significant impediments to the translational process in nanomedicine include safety concerns and socioeconomic difficulties. Although there are a few flaws to be addressed, such as how they integrate into therapeutic pathways, data consistency, and imaging methodologies, the promise is clear. UCNPs are set to become one of the most widely used nanoprobes in clinics in the near future.

## 8 Upconversion nanoplatform working as co-adjuncts in photodynamic therapy against resistant bacteria

Lanthanide-doped upconversion nanoparticles working as an energy donor in photodynamic therapy exhibit features that can be applied in resistant bacterial infections, once PDT does not request specific interaction with the etiological agent, ensuring gain advantage in a wide antibacterial spectrum and low acquired endurance. Furthermore, the property of upconversion nanoparticles to allow the generation of visible light under NIR (near infra-red) excitation, enable high deep tissue anti-infection treatment. In this sense, PDT can be associated with an antimicrobial agent for a synergic effect with these medical therapies. In the study developed by (Li et al., 2021), the group designed a hierarchical bio-inorganic nanoplatform based on NaYF_4_: Er,Yb@NaYF_4_ core-shell coated by dense silica and mesoporous silica to afford an efficient load of methylene blue as a photosensitizer and anti-microbial lysozyme, subsequently. To accomplish a high generation of ^1^O_2_, the methylene blue was combined precisely to preserve the monomeric structure as much as possible. This work also reports the nanoplatform could adequately annihilate the resistant bacteria *Staphylococcus aureus* infection localized in deep-tissue *in vivo*. Firstly, the works report the impact of the treatment based on single PDT *in vitro* indicated a devaluation of the bacteria. After that, the experiments performed on rats indicated exceptional effectiveness treatment of deep-tissue treatment.

## 9 Summary and perspectives

Even though some PS have already been approved by regulatory agencies, they still exhibit drawbacks that needs to be overcome. None of available PS presents a “magic bullet” containing all ideal characteristics required for a powerful PS. With its increased therapeutic efficacy, high space-time controllability, deep tissue penetration, and minimum invasion, UCNPs have significantly advanced the application of contemporary precision medicine in the life system during the past few decades. From experimental animals to human-level technical standard revisions, there is still a long way to go. The upconversion nanoparticles provide a remarkable opportunity to increase personalized medicine. Because of their stable surface modification, low toxicity, and specific application, UCNPs will become more competitive in the biological field. Nevertheless, considering the surface area of the nanomaterials based on rare earth hosts, the luminescence of rare earth is relatively lower compared to other phosphors such as Quantum Dots and organic dyes. Other fundamental parameters must be considered; to the best of our knowledge, the long-term biosafety of these materials is still unknown today. Biological processes such as pharmacodynamics and pharmacokinetics, toxicological parameters, and degradation call for additional research to mitigate the chronic biological cytotoxicity effects of rare earth nanomaterials.

It is worth mentioning that UCNPs can be used to transform nonimmunogenic tumors into immunogenic tumors which could allow an optimized PDT combined with immunotherapy (Ji et al., 2022). The combination of PDT with immunotherapy has been considered a good alternative treatment in managing recurrent metastatic tumors that are not eligible for surgery (Wang et al., 2022). Thus, all advantages described for UCNPs for PDT still can be useful for a smart prospect in clinical practice of PDT combined with immunotherapy, an emerging field in nanomaterials for PDT-driven cancer immunotherapy, especially in cases of tumor hypoxia (Wang et al., 2022) (Wang et al., 2022).

Based on the information presented in this review, progress must still be made so that all of the envisioned properties can be directly applied in the health sectors to reduce the global spread of cancer. Therefore, it is necessary to obtain a biologically compatible system, which is introduced into the human body composed of nanoparticles that are excited in the infrared in order to contribute to the generation of upconversion and consequently generate ROS and ^1^O_2_ to cause apoptosis. It would be interesting if this nanoparticle also had a molecule on its surface that chemically binds with a specific cancer-specific protein, allowing for a local treatment. In addition, if molecules that present signals that can be collected by magnetic resonance or electronic magnetic resonance were also located on the surfaces of these nanoparticles, the collection of information about that specific cancer would be increasingly known and, consequently, the understanding would be more in-depth for the resolution of a certain problem. We are confident that UCNPs advances have been opening new promising avenues to refine and improve the performance of PDT.
